# Preoperative vaginal preparation reduces postoperative systemic infectious complications in gynecologic surgery: a propensity score-matched retrospective cohort study

**DOI:** 10.3389/fcimb.2026.1847878

**Published:** 2026-08-20

**Authors:** Xiaojia Zheng, Liping Xu, Jin Peng, Qun Sun, Minhong Cai, Jing Chen, Pingping Wang, Yajun Bai, Qian Xiang

**Affiliations:** 1 Healthcare-associated Infection Control Center, Sichuan Academy of Medical Sciences, Sichuan Provincial People’s Hospital, School of Medicine, University of Electronic Science and Technology of China, Chengdu, Sichuan, China; 2 Hospital Infection Control Department, Sichuan Provincial People’s Hospital Pujiang Hospital-Pujiang People’s Hospital, Chengdu, Sichuan, China; 3 Hospital Infection Control Department, Sichuan Provincial People’s Hospital East Sichuan Hospital & Dazhou First People’s Hospital, Dazhou, Sichuan, China; 4 Chengdu Institute of Food Inspection; Irradiation Preservation Key Laboratory of Sichuan Province, Chengdu, China; 5 Department of Healthcare-associated Infection Management, Beijing Anzhen Nanchong Hospital of Capital Medical University & Nanchong Central Hospital, Nanchong, Sichuan, China

**Keywords:** doubly robust estimation, gynecological surgery, propensity score matching, systemic infectious complications, vaginal preparation

## Abstract

**Background:**

Postoperative systemic infectious complications (PSIC) impose a substantial burden on clinical resources following gynecologic surgery. While international guidelines recommend preoperative vaginal preparation, high-quality evidence supporting its efficacy against PSIC remains limited. Furthermore, uptake varies widely in practice, often reflecting healthcare reform priorities such as Diagnosis-Related Groups (DRG) and Diagnosis-Intervention Packet (DIP).

**Methods:**

We conducted a retrospective cohort study of 17,582 gynecologic surgery patients (January 2022–December 2024) at a southwestern Chinese tertiary hospital. Propensity score matching (PSM) produced 2,195 balanced pairs. The study outcome was the incidence of PSIC, assessed using an objective, EMR-based algorithmic definition.

**Results:**

Vaginal preparation reduced PSIC incidence by nearly half (1.28% vs. 2.19%; adjusted OR = 0.623, 95% CI: 0.385–0.919, p = 0.040). The protective effect was robust across pre-specified subgroups, with marked risk reductions observed among patients undergoing open surgery, those using prophylactic antibiotics, anemic patients, and non-diabetics.

**Conclusions:**

Preoperative vaginal preparation reduced PSIC risk in this real-world cohort. This intervention proved particularly valuable for specific patient and procedural subgroups. As a cost-effective measure, it warrants inclusion in routine preoperative protocols, even within efficiency-driven frameworks such as Enhanced Recovery After Surgery (ERAS) and DRG/DIP.

## Background

Gynecologic surgery addresses common female reproductive conditions, including uterine disorders, ovarian masses, ectopic pregnancy, and chronic pelvic inflammation (Hammer, 2015). Hysterectomy, oophorectomy, and laparoscopic procedures dominate surgical volumes, with minimally invasive approaches (including robotics) steadily replacing open surgery (Abdullah Agha et al., 2015; Gressel et al., 2020; Piątek et al., 2023). This shift has broadened overall surgical utilization.

Postoperative infections strain both outcomes and resources. Surgical site infections (SSIs) receive most research attention, but postoperative systemic infectious complications (PSIC) pose an equally serious threat—driving morbidity, mortality, and costs (ACOG, 2018) The American College of Obstetricians and Gynecologists (ACOG) and the Chinese Medical Association’s Gynecological Oncology Group (GOGMIPC) guidelines recommend preoperative vaginal preparation with povidone-iodine, chlorhexidine gluconate, or ethanol-based solutions for surgeries involving vaginal access (Xu et al., 2022). These recommendations carry 2B evidence levels, resting on observational data and expert consensus rather than rigorous trials.

Yet evidence remains conflicting. Several studies report no SSI reduction with vaginal preparation (Amstey and Jones, 1981; Cohen et al., 2023), and concerns about mucosal irritation or antiseptic allergies complicate routine adoption (Kjølhede et al., 2011). In China, uptake varies with healthcare policy. The 2019 National Health Commission metrics for tertiary hospitals and the 2021 DRG/DIP payment system emphasize efficiency and cost control—pressures that may truncate preoperative protocols and sideline vaginal preparation.

Most prior work targets SSIs specifically. PSIC data are scarce, and perioperative antibiotics may conceal true PSIC rates. We addressed these gaps using propensity score matching and doubly robust estimation to measure vaginal preparation’s impact on PSIC risk in gynecologic surgery, generating evidence for clinical practice that reconciles efficiency with patient safety in China’s evolving healthcare landscape.

## Methods

### Setting and patient population

We conducted this study at a tertiary teaching hospital in southwestern China. The Department of Gynecology performs approximately 7,000 procedures annually, handling benign and malignant conditions alike. Prophylactic antibiotics followed the National Health Commission’s 2015 Guidelines for Clinical Application of Antimicrobial Agents.

We retrospectively analyzed de-identified data from patients who underwent gynecologic surgery between January 2022 and December 2024. An automated Python pipeline (Python 3.9, pandas 1.4.0) extracted data from electronic medical records (EMR) and laboratory information systems (LIS), capturing demographics, clinical variables, diagnosis-related group (DRG) codes, laboratory results, operative details, and postoperative outcomes.

### Inclusion and exclusion criteria

Eligible patients met three criteria: age ≥18 years, available preoperative lab results (complete blood count, liver/renal panels), and documented height, weight, and comorbidities. We excluded patients having non-operating-room procedures or missing critical data.

### Intervention

Preoperative vaginal preparation consisted of vaginal swabbing or irrigation with 10% povidone-iodine, standardized as a single application lasting 3–5 minutes, performed by trained nurses on the day before or of surgery. We categorized patients into two groups based on documented EMR orders: vaginal preparation (intervention) or non-preparation (control). To prevent misclassification bias, the absence of vaginal preparation in the control group was rigorously cross-verified. Patients were assigned to the control group only if they had no documented physician orders, no corresponding nursing execution records (administration logs), and no itemized billing charges for this procedure.

### Study outcomes

The study outcome was PSIC. Due to the retrospective nature of this study, we employed a pragmatic, electronic medical record (EMR)-based surveillance definition adapted from the CDC/NHSN surveillance definitions for healthcare-associated infections (Rhee et al., 2016, 2017), alongside the Chinese National Diagnostic Criteria for Nosocomial Infections (Ministry of Health of the People’s Republic of China, 2001) and relevant studies (Copeland-Halperin et al., 2018; Tajima et al., 2021). Cases required all of the following within 30 days post-surgery: (1) fever >38.0 °C or hypothermia <36.0 °C for ≥2 consecutive days; (2) systemic signs of septicemia (toxic appearance, hypotension, or unexplained leukocytosis); (3) new or escalated systemic antibiotics; and (4) blood culture order. This syndromic definition captures microbiology-confirmed and clinically diagnosed infections. While some non-infectious SIRS cases may qualify, any deterioration severe enough to trigger blood cultures and empiric antibiotics carries equivalent resource burden—making this definition suitable for quantifying real-world costs. Incidence = (PSIC cases/total procedures) × 100%.

## Statistical analysis

### Propensity score matching

We estimated propensity scores via multivariable logistic regression (scikit-learn 1.0.2) using covariates: age, body mass index (BMI), ethnicity, white blood cell count (WBC), hemoglobin (HGB), platelets (PLT), creatinine (CREA), estimated glomerular filtration rate (eGFR), alanine aminotransferase (ALT), aspartate aminotransferase (AST), total bilirubin (TBIL), albumin (ALB), diabetes mellitus, hypertension, smoking, prophylactic antibiotics, American Society of Anesthesiologists score (ASA score), surgical approach, operative duration and 10 surgeon indicators (S1–S10 plus “Others”).

We performed 1:1 nearest-neighbor matching without replacement, with a stringent caliper width of 0.02 on the raw propensity score scale. Balance diagnostics were assessed via the absolute standardized mean difference (SMD) across all covariates, with SMD <0.1 indicating optimal balance. To serve as a sensitivity analysis for unmeasured or residual confounding post-matching, we subsequently applied doubly robust estimation.

### Outcome analyses

In the matched cohort, we compared PSIC incidence using McNemar’s test for paired binary outcomes, calculating crude matched-pairs odds ratios (ORs) and 95% confidence intervals (CIs).

To control residual confounding from covariates with SMD ≥0.1 post-PSM, we applied doubly robust estimation—combining inverse-probability weighting with outcome regression. We fitted a multivariable logistic model to the matched cohort, adjusting for vaginal preparation and residual-imbalance covariates, reporting Adjusted odds ratios (aORs) and 95% CIs.

### Subgroup analysis

Pre-specified subgroups tested vaginal preparation’s protective effect consistency. Stratification factors were: (1) surgical approach (minimally invasive, open, vaginal); (2) wound classification (Type I–II clean/clean-contaminated vs. Type III–IV contaminated/dirty); (3) diabetes; (4) hypertension; (5) prophylactic antibiotic use; (6) preoperative hemoglobin (<120 g/L vs. ≥120 g/L).

Within each subgroup, we quantified vaginal preparation–PSIC associations via unconditional logistic regression with Wald test, deriving subgroup-specific ORs, 95% CIs, and P-values. We assessed effect modification via likelihood ratio test comparing models with and without ‘Vaginal Preparation × Subgroup’ interaction, reporting P for interaction. All subgroup analyses used standard logistic regression within the matched cohort.

We applied two-sided tests with P <0.05 as the significance threshold. Analyses used Python 3.9 (scipy, statsmodels, scikit-learn) and R 4.3 (tables, cobalt).

## Results

### Study population and baseline characteristics

**Figure 1:**
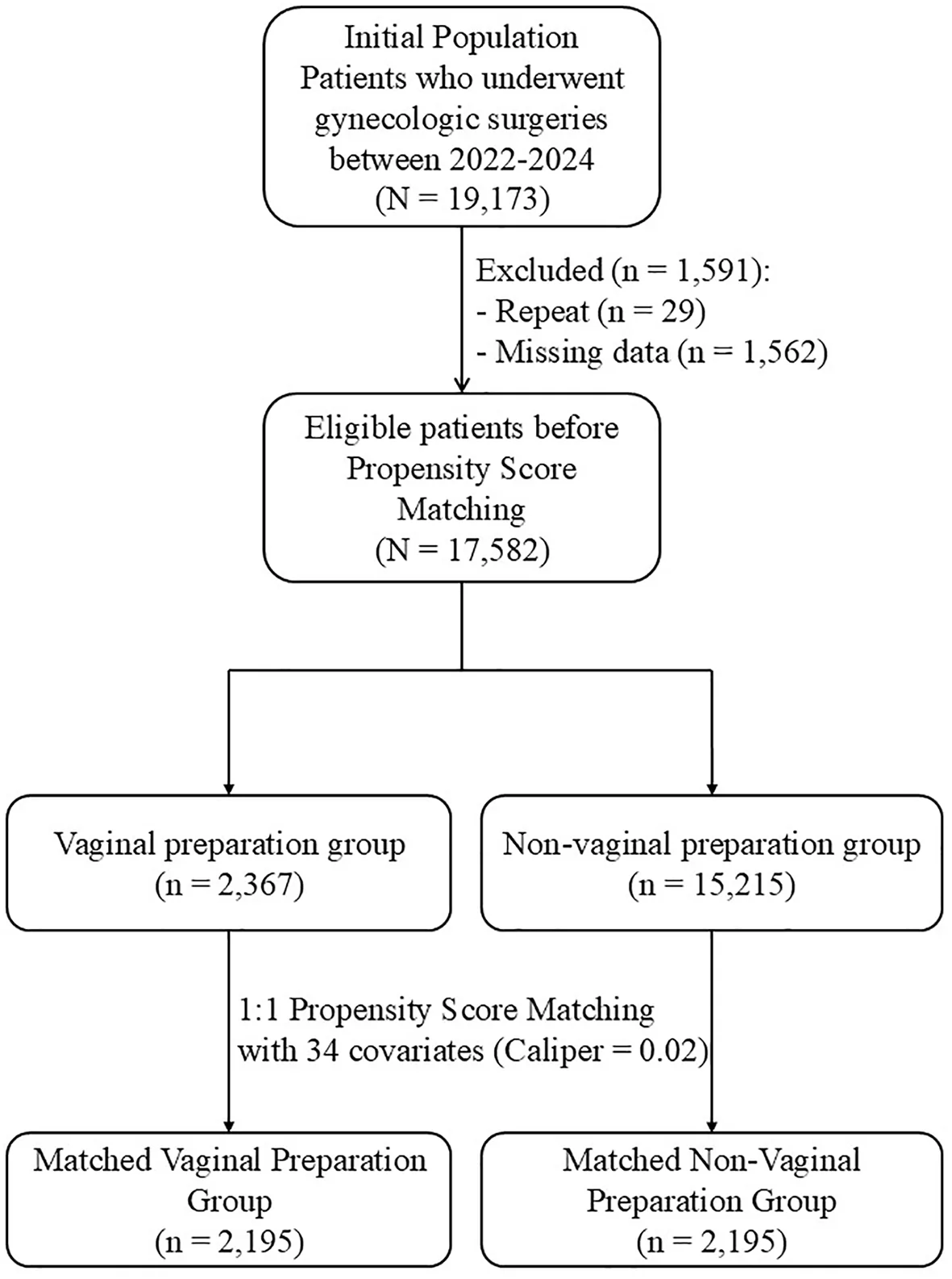
The participant selection process.

A total of 19,173 patients underwent gynecologic surgeries between January 2022 and December 2024. After applying exclusion criteria, 17,582 eligible patients entered propensity score matching (**Figure 1**).

**Figure 2:**
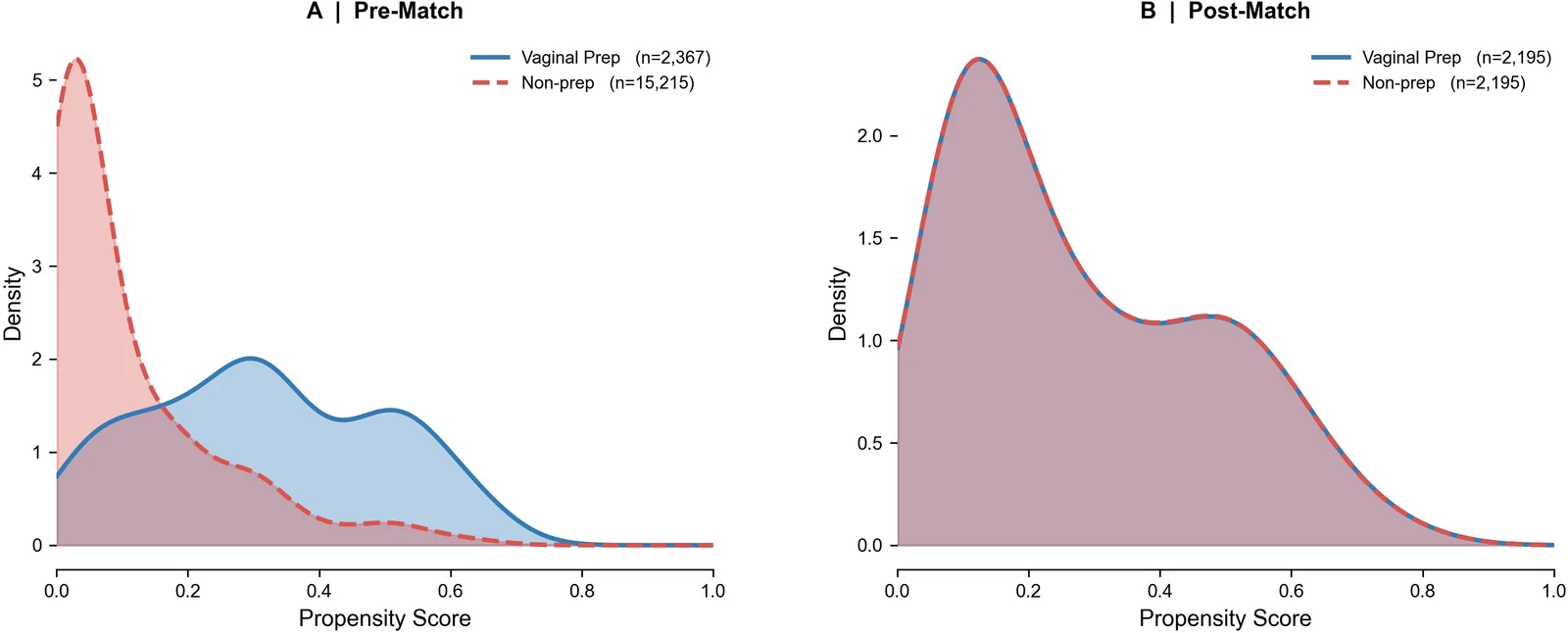
Kernel density plots pre- and post- propensity score matching.

**Figure 3:**
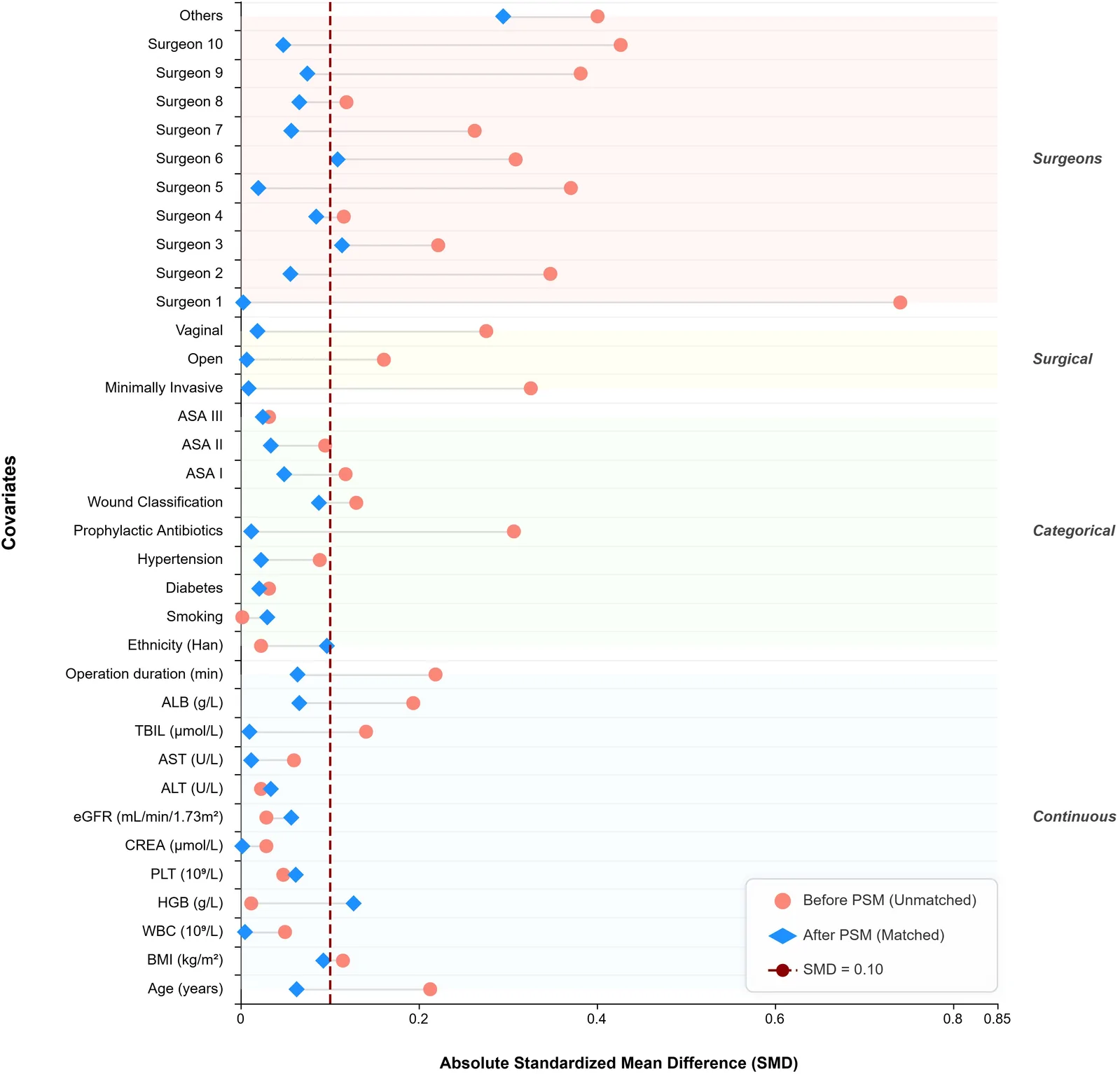
Standardized mean differences (SMD) of covariates pre- and post- propensity score matching.

Before matching, the vaginal preparation group (n=2,367) and non-preparation group (n=15,215) differed substantially in demographics, laboratory values, and surgical factors (**Table 1**). Propensity score distributions showed marked imbalance (**Figure 2A**). After 1:1 nearest-neighbor matching with covariates, we established 2,195 pairs (N = 4,390). Covariates achieved balance in the matched cohort (**Table 1**, **Figure 3**), and propensity score distributions overlapped well (**Figure 2B**).

**Table 1:** Baseline characteristics before and after propensity score matching.

Variable	Before PSM			After PSM		
	Vaginal prep (n=2,367)	Non-prep (n=15,215)	SMD	Vaginal prep (n=2,195)	Non-prep (n=2,195)	SMD
Continuous variables						
Age (years)	46.09 ± 12.60	43.40 ± 12.64	0.213	45.73 ± 12.50	44.91 ± 13.48	0.063
BMI (kg/m²)	23.26 ± 3.25	22.88 ± 3.28	0.115	23.22 ± 3.27	22.91 ± 3.26	0.093
WBC (10^9^/L)	9.76 ± 3.41	9.59 ± 3.43	0.050	9.71 ± 3.28	9.70 ± 3.34	0.005
HGB (g/L)	123.46 ± 64.08	124.24 ± 67.66	0.012	124.36 ± 63.10	116.74 ± 56.30	0.127
PLT (10^9^/L)	218.10 ± 73.76	214.62 ± 71.63	0.048	218.60 ± 73.35	214.01 ± 73.41	0.062
CREA (μmol/L)	98.03 ± 307.82	91.40 ± 210.64	0.029	97.31 ± 309.19	98.05 ± 339.23	0.002
eGFR (mL/min/1.73m²)	106.94 ± 17.20	107.43 ± 17.13	0.029	107.52 ± 16.56	106.59 ± 16.09	0.057
ALT (U/L)	20.53 ± 21.34	20.08 ± 18.52	0.023	19.85 ± 19.64	20.57 ± 22.70	0.034
AST (U/L)	25.03 ± 15.46	24.16 ± 14.18	0.060	24.77 ± 13.96	24.62 ± 12.74	0.012
TBIL (μmol/L)	14.57 ± 7.15	13.56 ± 7.16	0.141	14.50 ± 6.93	14.43 ± 7.40	0.010
ALB (g/L)	50.04 ± 36.49	59.76 ± 52.35	0.194	50.13 ± 36.64	52.71 ± 41.44	0.066
Operation duration (min)	93.97 ± 62.82	79.41 ± 66.88	0.219	91.61 ± 61.55	96.16 ± 78.67	0.064
Categorical variables						
Ethnicity (Han)	2,114 (89.3%)	13,697 (90.0%)	0.023	1,960 (89.3%)	1,890 (86.1%)	0.097
Smoking	1 (0.0%)	7 (0.0%)	0.002	1 (0.0%)	0 (0.0%)	0.030
Diabetes	102 (4.3%)	559 (3.7%)	0.032	95 (4.3%)	86 (3.9%)	0.021
Hypertension	294 (12.4%)	1,466 (9.6%)	0.089	264 (12.0%)	248 (11.3%)	0.023
Prophylactic Antibiotics	1,710 (72.2%)	8,760 (57.6%)	0.307	1,551 (70.7%)	1,539 (70.1%)	0.012
Wound Classification			0.130			0.088
Type I, II	2,113 (89.3%)	12,917 (84.9%)		1,948 (88.7%)	1,884 (85.8%)	0.096
Type III, IV	254 (10.7%)	2,298 (15.1%)		247 (11.3%)	311 (14.2%)	
ASA score						
ASA I	406 (17.2%)	3,324 (21.8%)	0.118	390 (17.8%)	432 (19.7%)	0.049
ASA II	1,852 (78.2%)	11,290 (74.2%)	0.095	1,704 (77.6%)	1,673 (76.2%)	0.034
ASA III	109 (4.6%)	601 (4.0%)	0.032	101 (4.6%)	90 (4.1%)	0.025
Surgical approach						
Minimally Invasive	1,768 (74.7%)	13,305 (87.4%)	0.326	1,681 (76.6%)	1,689 (76.9%)	0.009
Open	286 (12.1%)	1,113 (7.3%)	0.161	254 (11.6%)	259 (11.8%)	0.007
Vaginal	313 (13.2%)	797 (5.2%)	0.276	260 (11.8%)	247 (11.3%)	0.019
Surgeons						
1	843 (35.6%)	855 (5.6%)	0.741	674 (30.7%)	671 (30.6%)	0.003
2	26 (1.1%)	1,304 (8.6%)	0.348	26 (1.2%)	41 (1.9%)	0.056
3	313 (13.2%)	1,003 (6.6%)	0.222	310 (14.1%)	402 (18.3%)	0.114
4	145 (6.1%)	1,402 (9.2%)	0.116	145 (6.6%)	195 (8.9%)	0.085
5	26 (1.1%)	1,422 (9.3%)	0.371	26 (1.2%)	31 (1.4%)	0.020
6	19 (0.8%)	1,027 (6.7%)	0.309	20 (0.9%)	50 (2.3%)	0.109
7	88 (3.7%)	1,594 (10.5%)	0.263	88 (4.0%)	114 (5.2%)	0.057
8	153 (6.5%)	1,475 (9.7%)	0.119	153 (7.0%)	192 (8.7%)	0.066
9	15 (0.6%)	1,323 (8.7%)	0.382	15 (0.7%)	32 (1.5%)	0.075
10	27 (1.1%)	1,760 (11.6%)	0.427	27 (1.2%)	40 (1.8%)	0.048
Others	711 (30.0%)	2,050 (13.5%)	0.401	711 (32.4%)	427 (19.5%)	0.295

PSM, propensity score matching; SMD, standardized mean difference. Absolute SMD values are presented. In accordance with current statistical reporting guidelines for propensity score matched analyses, P-values are omitted as SMD < 0.1 indicates optimal balance independent of sample size.

### Postoperative systemic infectious complications (PSIC)

PSIC incidence was substantially lower with vaginal preparation (1.28% vs. 2.19%). The McNemar test yielded OR = 0.578 (95% CI: 0.361–0.925, p=0.022) (**Table 2**). After doubly robust adjustment for residual confounding, the aOR remained significant at 0.623 (95% CI: 0.385–0.919, p=0.040). Hemoglobin level and certain surgeons also showed independent associations—Surgeon 3 had a notably low OR of 0.443 (p=0.044) (**Table 2**).

**Table 2:** Effect of vaginal preparation on PSIC in the propensity score-matched cohort.

Outcomes & models	Vaginal prep (n = 2,195)	Non-prep (n = 2,195)	Odds ratio (95% CI)	P-value
Primary Outcome: PSIC, n (%)	28 (1.28%)	48 (2.19%)	–	–
Model 1: unadjusted analysis*				
Vaginal Preparation	–	Reference	0.578 (0.361–0.925)	0.022
Model 2: doubly robust estimation†				
Vaginal Preparation	–	Reference	0.623 (0.385–0.919)	0.040
Preoperative Hemoglobin (per 1 g/L)	-	-	0.998 (0.993–1.002)	0.301
Surgeon 3	–	Reference	0.443 (0.201–0.978)	0.044
Other Surgeons	-	Reference	0.609 (0.335–1.105)	0.103

PSIC, Postoperative Systemic Infectious Complication; CI, confidence interval; OR, odds ratio.

*Model 1 (Unadjusted Analysis) was calculated using McNemar's test for paired binary data.

†Model 2 (Doubly Robust Estimation) was adjusted for residual imbalances (Standardized Mean Difference > 0.1) after propensity score matching (1:1), including preoperative hemoglobin, Surgeon 3, and Other Surgeons. Note: Surgeon 6 was automatically excluded from the multivariable logistic regression model due to complete separation (0 PSIC events observed).

### Subgroup analysis

**Figure 4:**
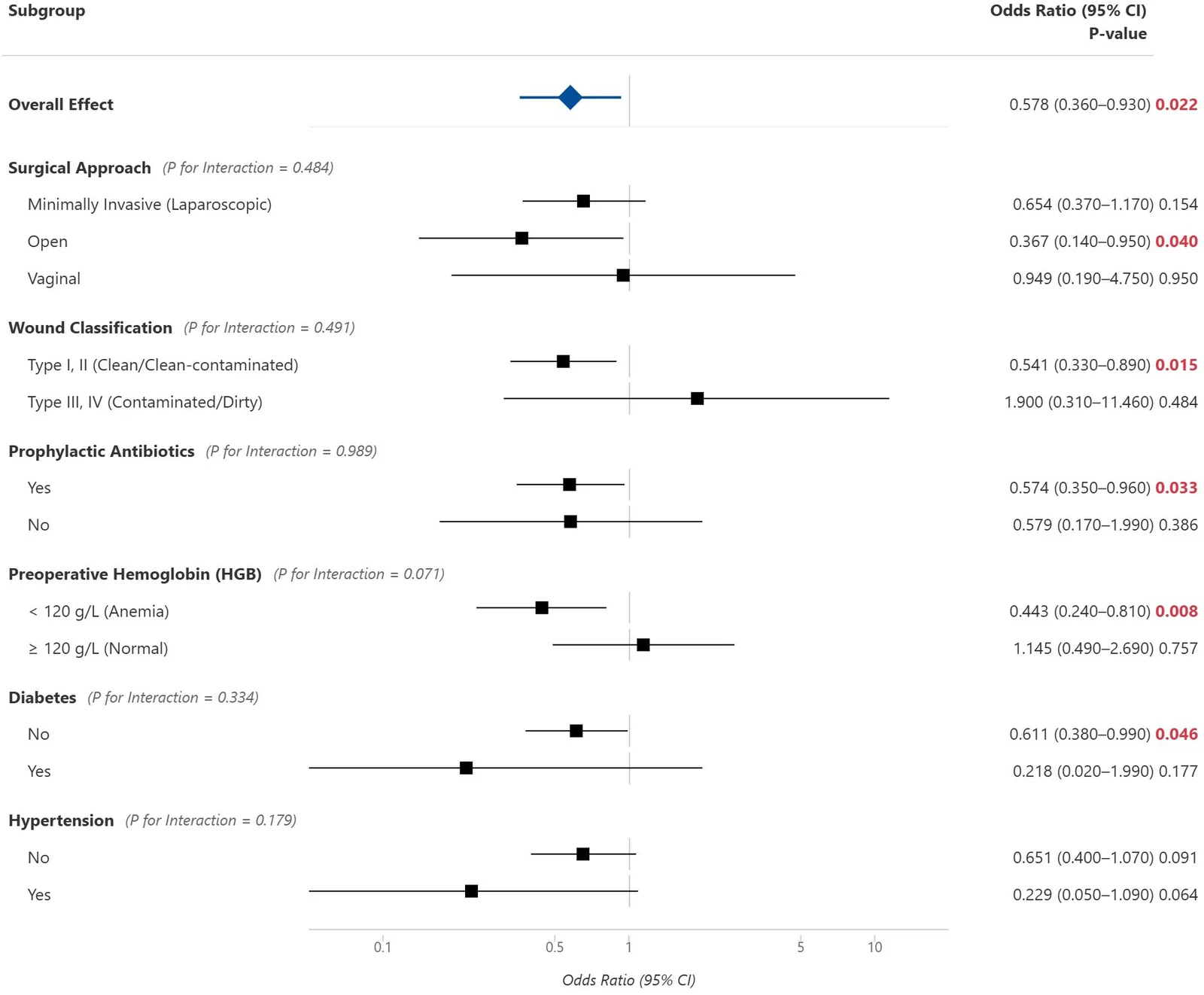
Subgroup analysis forest plot showing the association between vaginal preparation and PSIC.

Vaginal preparation’s protective effect held across most subgroups (**Figure 4**), with P for interaction >0.05 throughout. Significant risk reductions appeared in open surgery (OR = 0.367, 95% CI: 0.140–0.950, p=0.040), prophylactic antibiotic users (OR = 0.574, 95% CI: 0.350–0.960, p=0.033), hemoglobin <120 g/L (OR = 0.443, 95% CI: 0.240–0.810, p=0.008), and non-diabetics (OR = 0.611, 95% CI: 0.380–0.990, p=0.046).

## Discussion

Using propensity score matching and doubly robust estimation, this study developed a harmonized comparative cohort demonstrating that preoperative vaginal preparation significantly reduced postoperative systemic infectious complications (PSIC) in gynecologic surgery, with rates decreasing from 2.19% in the non-preparation group to 1.28% in the vaginal preparation group. The adjusted odds ratio (aOR) of 0.623 (95% CI: 0.385–0.919, p = 0.040) indicates a statistically significant protective effect. This is the first study to provide evidence for the clinical benefit of an intervention that reduces the risk of postoperative systemic infectious complications. Unlike previous studies, we systematically accounted for confounding variables through propensity score matching and further addressed residual confounding with a doubly robust approach, adding rigor to the methodology. This study went beyond traditional SSI surveillance by examining systemic infectious complications, an outcome rarely captured in prior literature (Hill et al., 2022).

Subgroup analyses confirmed vaginal preparation’s benefit across most strata. Open surgery patients showed the strongest protection (OR = 0.367, p=0.040), along with prophylactic antibiotic users (OR = 0.574, p=0.033), anemic patients (OR = 0.443, p=0.008), and non-diabetics (OR = 0.611, p=0.046). The mechanism likely involves reducing vaginal microbial load, which limits bacterial translocation into the bloodstream during surgery (Amstey and Jones, 1981; Buppasiri et al., 2004). Hill et al (Hill et al., 2022)showed greater bacterial suppression with antiseptic irrigation versus saline (p<0.001), supporting this pathway. Vaginal preparation also cuts bacterial migration risk in procedures with partial vaginal resection (Culligan et al., 2005). Our findings align with and strengthen prior research. Skeith et al. (2021) reported 38% infection reduction with vaginal preparation (p<0.05), and Buppasiri’s RCT (Buppasiri et al., 2004) found lower rates with povidone-iodine irrigation (8% vs. 19%, p<0.05). Kjølhede et al. found no benefit from chlorhexidine in abdominal hysterectomy (14.3% vs. 14.3%, OR = 0.92) or vaginal hysterectomy (13.5% vs. 7%) (Kjølhede et al., 2009, 2011). Based on our findings, we recommend routine preoperative vaginal preparation for gynecologic procedures when appropriate, especially given its demonstrated PSIC reduction across subgroups. This aligns with ERAS principles (Stone et al., 2021)and integrates well with DRG/DIP frameworks. We are also developing a patient-administered disposable antiseptic vaginal irrigation system to balance efficiency with preoperative preparation.

Importantly, our findings corroborate and extend the evidence base underpinning current international guidelines. The World Health Organization (WHO) global guidelines for the prevention of surgical site infections (SSI), the American College of Obstetricians and Gynecologists (ACOG), and the Royal College of Obstetricians and Gynecologists (RCOG) consistently recommend preoperative vaginal preparation with antiseptics. However, these recommendations are primarily predicated on the prevention of localized SSIs or vaginal cuff infections. By demonstrating a significant 40% reduction in systemic infectious complications (PSIC), our study provides crucial empirical support that the benefits of vaginal preparation extend far beyond the local surgical site to successfully mitigate severe, resource-intensive systemic infections.

Our results are especially relevant to Chinese practice. Current guidelines favor first- or second-generation cephalosporins for SSI prevention (National Health and Family Planning Commission & Chinese Medical Association, 2015), often with metronidazole for transvaginal procedures. These regimens have limited coverage against vaginal *Enterobacteriaceae*—the dominant PSIC pathogens in our population, with high cephalosporin resistance rates we previously found (Gong et al., 2025). Vaginal preparation offers a simple, cost-effective complement to existing antimicrobial strategies.

The ideal antiseptic remains unclear. Duong and Homewood (2024) found similar SSI rates for chlorhexidine and povidone-iodine (8.0% vs. 7.0%, p=0.12); Amstey (Amstey and Jones, 1981) found no difference between saline and povidone-iodine (8.0% vs. 7.0%, p=0.72); Culligan et al. (2005) favored chlorhexidine (p<0.05); Skeith et al. (2021) recommended povidone-iodine over chlorhexidine (3.0% vs. 4.3%, p=0.01). This inconsistency and lack of consensus question the need for more studies comparing antiseptic formulations and concentration for the prevention of infections.

This study has several limitations inherent to its design. First, as a single-center retrospective study, our findings may reflect institution-specific microbial epidemiologies and surgical practices, which could limit external validity across different healthcare settings. Second, while PSM and doubly robust estimation stringently controlled for observable confounders, and surgeon-specific fixed effects were included, unmeasured confounding—such as subtle variations in individual intraoperative techniques or exact tissue trauma extent—cannot be completely ruled out. Third, to prioritize optimal covariate balance and maximize internal validity, we employed a highly stringent matching caliper (0.02 on the raw propensity score scale) during PSM. Consequently, our matched cohort represents approximately 25% of the original eligible population. While this rigorous approach successfully minimized baseline confounding, it inevitably introduces potential selection bias and may limit the broader generalizability of our findings. Finally, our reliance on a pragmatic EMR-based surveillance algorithm may inadvertently capture non-infectious inflammatory phenomena. Systemic signs such as fever and leukocytosis can overlap with the normal physiological and metabolic responses to surgical trauma, postoperative stress, or non-infectious complications (e.g., atelectasis), potentially leading to an overestimation of true infectious complications. Furthermore, detailed acute-phase biomarkers (e.g., C-reactive protein, procalcitonin) and specific microbiological culture isolates were not consistently available across our retrospective database to definitively confirm all cases. Therefore, future prospective multicenter randomized controlled trials are warranted. These future studies should strictly standardize each step of the vaginal preparation technique (method, timing, and quality of application) to determine protocol-specific efficacy. Ideally, they should also incorporate standardized infection surveillance criteria, microbiological confirmation, and acute-phase biomarkers to rigorously distinguish true postoperative infections from expected sterile surgical inflammation.

## Conclusions

This study demonstrates that preoperative vaginal preparation significantly reduces the risk of postoperative systemic infectious complications in gynecologic surgery. Its protective effect was particularly notable in patients undergoing open surgery, those receiving prophylactic antibiotics, individuals with lower preoperative hemoglobin levels, and non-diabetic patients. This intervention offers a cost-effective strategy to enhance infection control practice, especially in settings where resistance to standard prophylactic antibiotics is a concern.

## Data Availability

The raw data supporting the conclusions of this article will be made available by the authors, without undue reservation.
